# Editorial: Case reports in thrombosis: 2025

**DOI:** 10.3389/fcvm.2026.1852681

**Published:** 2026-04-27

**Authors:** Luca Spiezia

**Affiliations:** First Chair of Internal Medicine, Department of Medicine, Padova University Hospital School of Medicine, Padova, Italy

**Keywords:** anticoagulant therapy, arterial thrombosis, case report, coagulopathic disorders, venous thrombosis

This editorial introduces a curated collection of articles published in Frontiers in Cardiovascular Medicine: Case Reports in Thrombosis: 2025. The manuscripts included in this Research Topic cover a broad and diverse range of subjects, spanning from genetic predispositions to venous thromboembolism to the clinical management of rare, complex, and potentially life-threatening comorbid conditions. Together, these contributions highlight recent advances, clinical challenges, and emerging perspectives in the field of thrombosis, offering valuable insights for both researchers and clinicians. We hope that readers will find this collection informative and practically relevant, supporting and enhancing decision-making in daily clinical practice. We would like to express our sincere gratitude to all the reviewers for their careful evaluations and constructive feedback, as well as to the Editorial Office staff for their continuous support and dedication throughout the publication process. Their contributions have been essential to the success and quality of this Research Topic. Below, we present the articles included in Frontiers in Cardiovascular Medicine: Case Reports in Thrombosis: 2025.

*Application of AngioJet Thrombectomy System in acute lower extremity deep vein thrombosis complicated by transplanted renal vein thrombosis (report of 2 cases)* by Shao et al.

Lower extremity deep vein thrombosis associated with transplanted renal vein thrombosis is a rare and severe post-surgical complication. This condition is characterized by venous obstruction, parenchymal edema, and a high risk of transplant failure. The authors describe a 41-year-old man and a 47-year-old man who had received an allogeneic kidney transplantation 15 and four years prior, respectively. Both patients presented with lower extremity pain and swelling. Color Doppler ultrasound in both cases showed thrombosis of the common femoral, iliac and transplanted kidney veins. The patients were treated with implantation of an inferior vena cava filter followed by mechanical thrombectomy using an AngioJet thrombus aspiration catheter. Residual stenosis in the common iliac vein required dilation with an ATLAS balloon. This report highlights that the AngioJet system is a safe and effective option in these patients to promptly remove the thrombus, restore blood flow and improve renal function without complications or recurrence. Furthermore, the authors underscore the importance or regular assessments of vascular patency in transplanted renal arteries and veins.

*Resolution of Subdural Hemorrhage Following Interventional Treatment of Superior Vena Cava Occlusion in a Hemodialysis Patient: A Case Report* by Lai et al.

Superior vena cava syndrome (SVCS) and non-traumatic subdural hemorrhage (SDH) are rare albeit life-threatening complications in hemodialysis patients. This case describes a 73-year-old hemodialysis male patient with end-stage renal disease who developed SVCS due to venous occlusion, later complicated by SDH. Interventional treatment with angioplasty and stent placement in the left brachiocephalic vein and SVC successfully resolved both conditions. The case suggests a pathophysiological link between venous congestion and SDH, as increased intracranial venous pressure may contribute to bleeding. Anticoagulation and hemodynamic fluctuations during dialysis may also worsen the condition. This report highlights that early treatment to relieve venous hypertension in SVCS is crucial to restore flow and prevent serious neurological complications in hemodialysis patients.

*Paradoxical embolism caused by totally implantable venous access port: A case* by Han et al.

Totally implantable venous access ports (TIVAPs) are widely used for chemotherapy and long-term infusions (1). Thrombosis associated with TIVAPs is uncommon and rarely leads to severe complications. This case describes a 58-year-old man with atrial fibrillation (AF) and patent foramen ovale (PFO) who developed paradoxical embolism and right atrial thrombosis 28 months after TIVAP implantation. The patient presented with dizziness and left lower extremity weakness. Imaging showed right temporal lobe infarction and a right atrial mass; he was treated with surgery and anticoagulation. This case highlights TIVAP risks and the importance of proper management, anticoagulation, and PFO screening in patients with AF and central venous catheter accesses.

*PROS1 (Cys228Tyr) missense mutation associated with mesenteric and pulmonary venous thromboembolism during the COVID-19 pandemic: a case report* by Huang et al.

Venous thromboembolism (VTE) may arise from genetic or acquired risk factors, including protein S deficiency (2). This report describes a 32-year-old man with mesenteric venous thrombosis and pulmonary embolism. He presented with pleuritic chest pain and mild fever 15 days after a confirmed COVID-19 diagnosis. Genetic testing revealed a PROS1 gene missense mutation. The patient had a previous episode of mesenteric thrombosis and several acquired risk factors such as obesity, physical inactivity, and inflammation; protein S activity was reduced. Despite initially receiving glucocorticoids and macrolide, symptoms worsened and D-dimer levels increased. A CT angiography confirmed acute pulmonary embolism. The patient initiated low-molecular-weight heparin and rivaroxaban; there was no recurrent VTE or bleeding events at one year. This case highlights the importance of effective therapeutic management in patients with inherited or acquired thrombophilias.

*Retrieval of an IVCF Retained for Over 6 Years* via *Femoral Venous Approach Using a Large-Bore Sheath: A Case Report and Literature Review* by Yuan et al.

Prolonged retention of inferior vena cava filters (IVCF) significantly increases the risk of complications such as thrombosis, migration, and vessel wall perforation (3). This case describes a patient who discontinued anticoagulation six years after IVCF implantation, and soon thereafter developed acute thrombosis of the IVC and iliac veins. Imaging revealed fibrotic adhesion, wall embedding, and filter strut perforation. Standard retrieval methods proved unsuccessful and more advanced techniques were necessary, including a loop snare approach to free the embedded hook — which initially failed as the filter was firmly incorporated into the vessel wall. Ultimately, successful removal was achieved using a retrograde femoral approach with a large vascular sheath. This case highlights advanced retrieval strategies for long-term IVCFs causing life-threatening complications.

*Tissue Prolapse-Induced Acute Stent Thrombosis: A Case Report and Intravascular Imaging-Based Insight* by Zhang et al.

Acute stent thrombosis (AST) is a rare but severe complication that may occur within 24 h of percutaneous coronary intervention (PCI), usually due to inadequate antiplatelet therapy, stent underexpansion or malapposition (4). This case describes a patient with ST-elevation myocardial infarction who developed recurrent ischemia one hour after PCI. Imaging revealed stent occlusion, and intravascular ultrasound identified significant tissue prolapse as the underlying cause, despite correct stent placement and adequate anticoagulation. Balloon angioplasty was ineffective, and the patient required the implantation of a second stent, which resolved the issue. This report highlights that clinicians should suspect tissue prolapse in patients with AST and no signs of dissection and malapposition. Furthermore, the authors underscore the importance of intravascular imaging for accurate diagnosis and timely management.

*Dilated Thoracoabdominal and Epigastric Veins in a Hemodialysis Patient with SVC Occlusion: Case Report and Literature Review* by Zhou et al.

Long-term use of hemodialysis catheters has been associated with an increased incidence of superior vena cava (SVC) obstruction. This case presents a 50-year-old hemodialysis patient admitted for markedly dilated thoracoabdominal and superficial epigastric veins. Digital subtraction angiography showed a complete superior vena cava (SVC) occlusion. Color Doppler ultrasound detected no AVF stenosis; no increased venous pressure was observed during multiple hemodialysis sessions. The patient presented no swelling, pain, AV access dysfunction, neurological or respiratory symptoms. Therefore, the authors opted for a conservative management with no immediate intervention. After five years of monthly-to-quarterly follow-up visits, the patient's left forearm AVF and compensated epigastric veins could be used for hemodialysis access. This case highlights the challenges of managing central venous obstruction, showing that conservative, patient-centered approaches can achieve excellent long-term outcomes with effective collateral compensation.

*Multisystem Embolism in Hereditary Protein C Deficiency with Patent Foramen Ovale: A Case Report* by Lihua *Chen* et al.

Hereditary protein C deficiency (HPCD) is a rare thrombophilia that increases the risk of venous thrombosis (2). A patent foramen ovale (PFO) with right-to-left shunt can enable paradoxical embolism. We report a 29-year-old woman with sudden altered consciousness. The patient had no traditional cerebrovascular risk factors except oral contraceptive use. Imaging revealed basilar artery occlusion, pulmonary embolism, and bilateral iliac vein thrombosis, and confirmed the presence of PFO with a significant right-to-left shunt. Laboratory tests showed reduced protein C activity and a pathogenic PROC mutation. She was treated with mechanical thrombectomy, IVC filter placement, PFO closure, and lifelong anticoagulation with rivaroxaban. At one-year follow-up, she had fully recovered without recurrence, hence the need for tailored management for better outcomes. This case highlights the significant risk of multisystem thromboembolic events in patients with HPCD compounded by PFO. Furthermore, the authors argue that clinicians should thoroughly investigate the presence of both conditions in patients with unexplained multisystem embolism.

*Carotid Artery Stenting in JAK2 V617F Positive Essential Thrombocythemia with Symptomatic Internal Carotid Artery Stenosis: A Case Report* by Du et al.

Essential thrombocythemia (ET) is a myeloproliferative neoplasm characterized by abnormal megakaryocyte proliferation and markedly increased platelet production — linked to the JAK2 V617F mutation in 50%–60% of patients (5). It is associated with significantly increased thrombotic and hemorrhagic risk. We report a 66-year-old man with previously diagnosed JAK2 V617F-positive ET who presented with transient slurred speech and right-sided facial droop. Imaging showed acute cerebral infarction and right internal carotid artery stenosis. The patient successfully underwent carotid stenting with aggressive perioperative platelet and inflammation management using hydroxyurea, aspirin, and ticagrelor. This case highlights the vital need for thorough perioperative management, pharmacogenomics-based antiplatelet therapy and multidisciplinary management in high-risk ET patients undergoing carotid artery stenting.

*Management of intracardiac thrombosis in newborns: a case series and a narrative review of the literature* by De Rose et al.

Neonatal intracardiac thrombosis (ICT) is a rare but potentially life-threatening condition associated with increased morbidity and mortality in term and preterm infants. This study reports two cases: an infant born at 37 gestational weeks with atrial thrombus at the fossa ovalis detected on transthoracic echocardiography within one hour of birth; and an infant born at 36 gestational weeks who developed left atrial thrombus (day 7) after resuscitation at birth and severe anemia requiring two blood transfusions. Both were successfully treated with low-molecular-weight heparin (LMWH) without complications. Critically ill neonates require high level of suspicion and close monitoring for thromboembolic events, and management decisions are challenging. Supportive care addressing underlying conditions (e.g., anemia, infections), combined with individualized anticoagulation, can yield favorable outcomes. This case highlights the utmost importance of prompt diagnosis and multidisciplinary care for the effective management of ICT in newborns.

*A patient with sudden pulmonary embolism and stroke after total hysterectomy and bilateral salpingooophorectomy was diagnosed with patent foramen ovale: case report and review* by Zhao et al.

A 75-year-old woman underwent laparoscopic hysterectomy with bilateral salpingo-oophorectomy to treat uterine fibroids. On the first postoperative day, she experienced sudden convulsions and loss of consciousness, with investigations revealing pulmonary embolism and partial thrombosis in the right lower extremity veins. Anticoagulant therapy was initiated. On postoperative day three, she showed agitation and a positive right Babinski sign; a brain CT scan confirmed a cerebral infarction. The unusual sequence of events from pulmonary to cerebral ischemic events led the clinicians to suspect paradoxical embolism. Transesophageal echocardiography with bubble study confirmed a patent foramen ovale (PFO). This case underscores the importance of investigating underlying causes in atypical postoperative thromboembolic presentations. Furthermore, the authors propose that early diagnosis of PFO and subsequent preventative measures may help mitigate the increased postoperative risk of stroke.

*Concurrent Hemorrhage and Thrombosis: A Case Report of Hemoptysis from Pulmonary Vein Stenosis with Left Ventricular Thrombus* by Chen et al.

Pulmonary vein stenosis (PVS) is a severe postoperative complication of cardiac ablation to treat atrial fibrillation. Furthermore, dilated cardiomyopathy is associated with a significantly increased risk of left ventricular thrombus (LVT). This case describes a 46-year-old man with dilated cardiomyopathy and prior ablation for atrial fibrillation who presented with hemoptysis and dyspnea. Imaging revealed concomitant left superior PVS and LVT, creating a conflict between the need to control bleeding and anticoagulation. The clinicians opted for a staged therapeutic approach. First, urgent balloon angioplasty with stent placement to resolve the obstruction and hemoptysis, followed by anticoagulation with rivaroxaban and clopidogrel. At six months, symptoms of hemoptysis and dyspnea resolved completely and the thrombus was dissolved; albeit in-stent re-occlusion occurred. This case highlights the delicate balance between managing thrombosis and bleeding in patients with rare concomitant PVS and LVT. The authors propose that prioritizing the recanalization of the pulmonary vein before initiating anticoagulation may yield more favorable, long-term outcomes.

*Pneumomediastinum and Pulmonary Embolism in a 19-Year-Old with Focal Segmental Glomerulosclerosis: A Rare Double-Complication of Corticosteroid Therapy* by Rotaru and Achim.

Focal Segmental Glomerulosclerosis (FSGS) is a rare immune-mediated glomerulonephropathy. It is a common cause of nephrotic syndrome and end-stage renal disease, often treated with corticosteroids (6). This case describes a 19-year-old man previously diagnosed with FSGS secondary to anabolic steroid use and receiving glucocorticoid therapy. He presented with acute respiratory distress and neck swelling, and was subsequently diagnosed with pneumomediastinum, saddle pulmonary embolism and DVT. This rare combination highlights the complexities of managing corticosteroid therapy in young patients, balancing efficacy against potentially life-threatening complications. The patient had a hypercoagulable profile stemming from the combination of corticosteroid therapy, testosterone-induced polycythemia and nephrotic syndrome. This case underscores the importance of closely monitoring this subset of patients, and balancing anticoagulant therapy with corticosteroids to minimize adverse events.

*A Case of Severe Viral Pneumonia Complicated by Pulmonary Embolism Treated with Extracorporeal Membrane Oxygenation Combined with Interventional Thrombectomy* by Wang et al.

H1N1 influenza is a known cause of pneumonia and acute respiratory distress syndrome (ARDS). This case describes a 70-year-old man with severe H1N1 influenza pneumonia who developed intermediate-high risk pulmonary embolism (PE). He presented with dyspnea and fever, and chest CT showed bilateral interstitial infiltrates, while PCR confirmed H1N1 infection. Despite receiving antivirals, antibiotics, and respiratory support, he developed refractory hypoxemia and rising D-dimer levels. A CT pulmonary angiography confirmed PE, and he underwent catheter-directed thrombolysis (CDT) under venoarterial ECMO support. This approach led to rapid hemodynamic and respiratory improvement, resulting in full recovery and discharge. The significant overlap in clinical manifestations between ARDS and PE may explain the underrecognition of the latter in these patients. This case emphasizes the importance of suspecting PE in patients with severe non-responding pneumonia, and highlights ECMO-assisted CDT as an effective rescue therapy.

*Case Report: Upper Extremity Deep Vein Thrombosis Revealing an Occult Invasive Ductal Breast Carcinoma* by Chiorescu et al.

Upper extremity deep vein thrombosis (DVT) is a rare albeit clinically significant condition (7). This case describes a 66-year-old woman who developed extensive left upper extremity DVT after carpal tunnel surgery. Imaging revealed tumor infiltration of the subclavian vessels causing thrombosis and arterial stenosis. Despite elevated tumor markers, initial breast imaging was negative. A chest CT-guided biopsy confirmed invasive lobular breast carcinoma. The case is notable for its atypical presentation, with breast cancer mimicking carpal tunnel syndrome and lacking detectable breast lesions on mammography or ultrasonography, despite infiltrating the axillary vasculature. Management included anticoagulation and individualized oncologic therapy. This report highlights that upper extremity DVT is frequently associated with occult cancer and may in fact be its earliest clinical manifestation. Early diagnosis and timely patient-tailored anticoagulation and oncologic treatment are paramount for better outcomes.

*Hyperfibrinolysis during intra-aortic balloon pump support: A case report on targeted tranexamic acid therapy* by Dong et al.

Patients with end-stage heart failure often require bridging therapy with intra-aortic balloon pump (IABP) to maintain hemodynamic stability. Although minimally invasive, IABP is associated with complex coagulation abnormalities (8). This case describes a 49-year-old man awaiting heart transplantation who developed IABP-induced secondary hyperfibrinolysis due to infection, and hemodynamic instability. He presented with elevated D-dimer levels and persistent bleeding. Laboratory findings confirmed increased fibrinolytic activity. Treatment with intravenous tranexamic acid effectively controlled bleeding and corrected the imbalance without causing thrombosis, allowing successful bridging to transplantation. This case highlights the need to recognize hyperfibrinolysis in patients with end-stage heart failure, especially those undergoing mechanical circulatory support.

*Thromboembolism in the Left Main Coronary Artery of a Patient With Membranous Nephropathy: A Case Report* by Wang et al.

Membranous nephropathy (MN) is characterized by a thickening of glomerular capillary walls due to the deposition of immune complexes. It is a major cause of nephrotic syndrome and is associated with hypercoagulability and hypercholesterolemia (9). This report describes the fatal case of a 48-year-old man with previously diagnosed MN who presented with a 17-hour history of chest pain. An emergency coronary angiography showed acute thrombosis with complete occlusion of the left main coronary artery. Despite thrombus aspiration and stent placement, the patient died from refractory cardiogenic shock. This case highlights that MN-related hypercoagulability may also cause fatal arterial thrombosis in rare cases. Clinicians should remain vigilant in the presence of MN patients lamenting chest pain with no other apparent risk factors, and even consider prophylactic anticoagulation for high-risk MN patients.

*Pathogenic interplay between markedly elevated plasma Lipoprotein(a) levels and prothrombotic mechanisms: a case report* by Biolo et al.

Lipoprotein(a) [Lp(a)] is a naturally occurring molecule whose levels may significantly the risk of developing atherosclerotic cardiovascular disease (10). Its role in promoting thrombosis is not yet fully understood. This case report describes a 64-year-old woman with extremely elevated Lp(a) levels and a history of cardiovascular events, including triple coronary artery bypass graft. She presented with difficult-to-control hypercholesterolemia despite maximally tolerated lipid-lowering and antiplatelet therapy. Functional tests showed a hypercoagulable state, including increased thrombin generation and platelet hyperreactivity. The patient received anticoagulation and low-intensity statin therapy (due to documented intolerance to higher doses). She was discharged and readmitted multiple time over the course of eight months for recurrent thromboembolic events. Planned lipoprotein apheresis was terminated early due to the onset of vasovagal syncope, and the patient died ten days later from myocardial infarction. This case highlights the possible association between markedly elevated Lp(a) levels and thrombotic risk, highlighting the need for tailored antithrombotic strategies in these patients.
